# Characterization of specimens for quality control of malaria rapid diagnostic tests: what is the best indicator for performance?

**DOI:** 10.1186/1475-2875-13-S1-P76

**Published:** 2014-09-22

**Authors:** Roxanne Rees-Channer, Jane Cunningham, Peter Chiodini, John Barnwell, Jeffery Glenn, Iveth Gonzalez, David Bell, Sandra Incardona

**Affiliations:** 1 Hospital for Tropical Diseases, London, UK; 2 GMP World Health Organisation, Geneva, Switzerland; 3 Center for Disease Control and Prevention, Atlanta, USA; 4 Foundation for Innovative New Diagnostics, Geneva, Switzerland; 5 Greater Good/Intellectual Ventures Lab, Seattle, USA

## 

Malaria rapid diagnostic tests (RDTs) are now considered an acceptable alternative for parasite based diagnosis in the absence of adequate quality microscopy. Selection of the most appropriate RDT among the sheer number of tests available commercially has long been a challenge, raising the need to objectively assess and compare test performance. In order to facilitate RDT procurement and stimulate improvement of the quality of RDTs, methods were developed for the collection and characterization of geographically diverse, malaria infected clinical blood specimens to set up a specimen bank for the evaluation of malaria RDTs, as part of the WHO Malaria RDT Product Testing programme. Parasite positive samples were diluted to low (200 p/ul) and higher (2000 p/ul) parasite densities and used to provide comparative data on the performance of RDT lots submitted by manufacturers. Only samples containing a single species and producing consistent antigen quantification results were eligible for inclusion in the WHO Malaria Specimen Bank. At both 200 and 2000 p/μl, a large variation in antigen concentrations was found, especially for *P. falciparum* HRP2, and to a lesser extent for pLDH and aldolase (both in *P. falciparum* and *P. vivax* samples); a trait that was independent of geographical origin. Possible reasons for this include the duration of infection at the time of blood collection and principally, differences in antigen expression between different isolates and stages of parasites. It is critical that performance evaluation panels are standardised at a parasite density being not only clinically relevant but also being close to the test detection limit to detect differences in test performance. Given the significant fluctuation in antigen concentrations at a given parasitaemia, appropriate sample sizes are required to cover that range and challenge the RDTs at the low end of this range of antigen concentrations. Antigen concentrations that are relevant for improvements in target sensitivity for RDTs are still to be determined for samples with parasite densities below 200 p/μl.

